# Mask Use and Ventilation Improvements to Reduce COVID-19 Incidence in Elementary Schools — Georgia, November 16–December 11, 2020

**DOI:** 10.15585/mmwr.mm7021e1

**Published:** 2021-05-28

**Authors:** Jenna Gettings, Michaila Czarnik, Elana Morris, Elizabeth Haller, Angela M. Thompson-Paul, Catherine Rasberry, Tatiana M. Lanzieri, Jennifer Smith-Grant, Tiffiany Michelle Aholou, Ebony Thomas, Cherie Drenzek, Duncan MacKellar

**Affiliations:** ^1^CDC COVID-19 Response Team; ^2^Georgia Department of Public Health; ^3^Epidemic Intelligence Service, CDC; ^4^4ES Corporation, San Antonio, Texas.

To meet the educational, physical, social, and emotional needs of children, many U.S. schools opened for in-person learning during fall 2020 by implementing strategies to prevent transmission of SARS-CoV-2, the virus that causes COVID-19 (*1*,*2*). To date, there have been no U.S. studies comparing COVID-19 incidence in schools that varied in implementing recommended prevention strategies, including mask requirements and ventilation improvements* (*2*). Using data from Georgia kindergarten through grade 5 (K–5) schools that opened for in-person learning during fall 2020, CDC and the Georgia Department of Public Health (GDPH) assessed the impact of school-level prevention strategies on incidence of COVID-19 among students and staff members before the availability of COVID-19 vaccines.^†^ Among 169 K–5 schools that participated in a survey on prevention strategies and reported COVID-19 cases during November 16–December 11, 2020, COVID-19 incidence was 3.08 cases among students and staff members per 500 enrolled students.^§^ Adjusting for county-level incidence, COVID-19 incidence was 37% lower in schools that required teachers and staff members to use masks, and 39% lower in schools that improved ventilation, compared with schools that did not use these prevention strategies. Ventilation strategies associated with lower school incidence included methods to dilute airborne particles alone by opening windows, opening doors, or using fans (35% lower incidence), or in combination with methods to filter airborne particles with high-efficiency particulate absorbing (HEPA) filtration with or without purification with ultraviolet germicidal irradiation (UVGI) (48% lower incidence). Multiple strategies should be implemented to prevent transmission of SARS-CoV-2 in schools (*2*); mask requirements for teachers and staff members and improved ventilation are important strategies that elementary schools could implement as part of a multicomponent approach to provide safer, in-person learning environments. Universal and correct mask use is still recommended by CDC for adults and children in schools regardless of vaccination status (*2*).

Beginning in fall 2020, many Georgia schools opened for in-person learning. At that time, GDPH required all Georgia schools to submit weekly data on the aggregate number of COVID-19 cases among students and staff members.^¶^ School-associated cases were self-reported by parents and guardians of students, or staff members, or those reported by local public health officials. On November 16, 2020, the Georgia Department of Education and local health districts emailed an online survey on behalf of CDC and GDPH to all Georgia public K–5 school district superintendents (1,321 schools) and private school leaders (140 schools) to assess school and student characteristics and COVID-19 prevention strategies implemented at the time of the survey. Weekly reminders were sent for 3 additional weeks. Surveys were completed by principals (67.0%), nurses (12.0%), assistant principals (4.7%), or other school representatives (16.4%). School characteristics assessed included school type,** urban-rural classification,^††^ and instructional model.^§§^ Student characteristics assessed included racial/ethnic distribution^¶¶^ and percentages of students who received in-person instruction. Prevention strategies assessed included mask requirements for teachers, staff members, and students; ventilation improvements***; physical distancing of desks (≥6 ft apart); barriers on student desks; class size (number of students in a classroom); cohort size (small groups of students who stay together throughout the day during in-person learning); and number and locations of available handwashing stations. Survey data were collected by CDC and stored in REDCap (version 9.7; Vanderbilt University).

Reported COVID-19 cases submitted to GDPH and online survey data collected during November 16–December 11, 2020, were linked by school to examine associations between prevention strategies and COVID-19 incidence, defined as number of cases among students and staff members per 500 enrolled students during the study period. Rate ratios (RRs) and 95% confidence intervals (CIs) were estimated with negative binomial regression models, adjusted for county-level 7-day incidence (cases per 100,000 population) on December 1, 2020.^†††^ Rate ratios with 95% CIs excluding 1.0 were considered statistically significant. Analyses were conducted in R (version 4.0.2; The R Foundation). This activity was reviewed by CDC and was conducted consistent with applicable federal law and CDC policy.^§§§^

Representatives from 169 (11.6%) of 1,461 schools in 51 (32.1%) of 159 Georgia counties (median = two schools per county) completed the survey and also had available COVID-19 case data (Figure).^¶¶¶^ Schools reporting 100% virtual learning were excluded. Among the 169 schools, 162 (95.9%) were public, representing 47 (26.0%) of 181 public school districts in Georgia (median = two schools per district). Schools had a median of 532 enrolled students (attending virtually and in-person), 91.1% were publicly funded, 71.0% were located in metropolitan areas, and 82.2% used hybrid learning (Table 1). Median class size was 19.0 students (interquartile range [IQR] = 15.0–21.0); median cohort size was 20.0 students (IQR = 15.0–21.0). Among all schools, the proportion of students receiving at least some in-person instruction ranged from 8.5% to 100% (median = 84.7%); 3.0%–100% (median = 64.0%) were eligible for free or reduced-cost meal plans, and approximately one half of students were White (median = 55.1%), followed by Black (median = 17.0%), Hispanic (median = 9.0%), multiracial (median = 4.5%), and Asian (median = 1.0%).****

**FIGURE:**
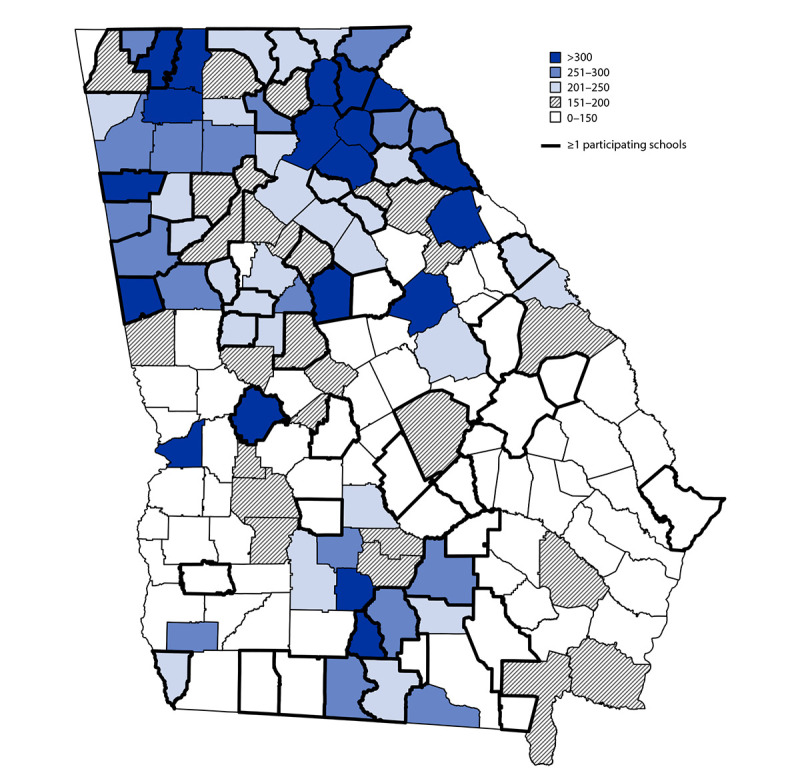
County-level COVID-19 incidence* on December 1, 2020, among counties with one or more participating elementary schools^†^ and counties without participating schools — Georgia, November 16−December 11, 2020 **Abbreviations:** GDPH = Georgia Department of Public Health; K–5 = kindergarten through grade 5. * County incidence was calculated as the 7-day cumulative sum of COVID-19 cases reported to GDPH divided by the county population multiplied by 100,000 on December 1, 2020. Population estimates for 2019 were provided by the Annual Estimates of the Resident Population for Counties in Georgia from April 1, 2010, to July 1, 2019. ^†^ GDPH and Georgia Department of Education contacted all public Georgia K−5 superintendents (1,321 schools) and private school leaders (140 schools). Representatives from 169 schools with available case data completed the survey (11.6% of schools contacted).

**TABLE 1:** COVID-19 incidence* and rate ratios in 169 elementary schools,**^†^** by county COVID-19 incidence, school characteristics, and COVID-19 prevention strategies — Georgia, November 16–December 11, 2020

Characteristic	No. (%) of schools	No. of enrolled students	No. of cases^§^	Cases per 500 students enrolled (95% CI)	RR^¶^ (95% CI)
**Total**	**169 (100)**	**91,893**	**566**	**3.08 (2.84–3.34)**	**—**
County COVID-19 incidence**					
0–150	25 (14.8)	12,358	52	2.10 (1.61–2.76)	Ref
151–200	54 (32.0)	32,399	169	2.61 (2.24–3.03)	1.21 (0.75–1.96)
201–250	45 (26.6)	24,482	106	2.16 (1.79–2.62)	1.00 (0.60–1.66)
251–300	21 (12.4)	11,556	122	5.28 (4.42–6.30)	2.55 (1.47–4.47)
>300	24 (14.2)	11,098	117	5.27 (4.40–6.31)	2.26 (1.32–3.88)
**School type**					
Public	154 (91.1)	86,878	536	3.08 (2.84–3.36)	Ref
Public charter/Magnet/Alternative	8 (4.7)	4,645	27	2.91 (2.00–4.22)	0.97 (0.50–1.97)
Private/Parochial/Independent	7 (4.1)	370	3	4.05 (1.38–11.78)	1.46 (0.31–5.33)
**Urban–rural setting** ^††^					
Metropolitan	120 (71.0)	65,501	386	2.95 (2.67–3.25)	Ref
Nonmetropolitan	49 (29.0)	26,392	180	3.41 (2.95–3.94)	1.14 (0.83–1.58)
**Instructional model** ^§§^					
100% in-person	30 (17.8)	14,538	106	3.65 (3.02–4.41)	Ref
Hybrid	139 (82.2)	77,355	460	2.97 (2.71–3.26)	0.91 (0.60–1.36)
**Mask requirements for teachers and staff members** ^¶¶^					
Optional	57 (33.7)	29,881	264	4.42 (3.92–4.98)	Ref
Required	110 (65.1)	61,190	298	2.44 (2.17–2.73)	0.63 (0.47–0.85)
**Mask requirements for students**					
Optional	82 (48.5)	42,761	326	3.81 (3.42–4.25)	Ref
Required	87 (51.5)	49,132	240	2.44 (2.15–2.77)	0.79 (0.50–1.08)
**Flexible medical leave policies for teachers**					
Not offered	31 (18.3)	17,194	137	3.98 (3.37–4.71)	Ref
Offered	138 (81.7)	74,699	429	2.87 (2.61–3.16)	0.81 (0.56–1.17)
**Ventilation improvements**					
No***	37 (21.9)	21,844	183	4.19 (3.63–4.84)	Ref
Yes	87 (51.5)	44,771	234	2.61 (2.30–2.97)	0.61 (0.43–0.87)
Don’t know	45 (26.6)	25,278	149	2.95 (2.51–3.46)	0.63 (0.42–0.95)
**Desks or tables separated *≥*6 ft**					
Some/No classrooms	137 (81.1)	76,348	472	3.09 (2.83–3.38)	Ref
All classrooms	32 (18.9)	15,545	94	3.02 (2.47–3.70)	0.97 (0.66–1.45)
**Desks or tables with barriers**					
Some/No classrooms	131 (77.5)	71,163	445	3.13 (2.85–3.43)	Ref
All classrooms	38 (22.5)	20,730	121	2.92 (2.44–3.48)	0.98 (0.69–1.41)
**Students per classroom, median (IQR)**	19 (15–21)	—	—	—	1.02 (0.98–1.06)
**Cohort size,^†††^ median (IQR)**	20 (15–21)	—	—	—	1.00 (1.00–1.00)
**Handwashing stations, median (IQR)**	9 (8–9)	—	—	—	0.88 (0.76–1.01)

**Abbreviations:** CI = confidence interval; IQR = interquartile range; GDPH = Georgia Department of Public Health; K–5 = kindergarten through grade 5; RR = rate ratio; Ref = referent.

* Case incidence in schools was calculated as the sum of cases reported to GDPH during November 16–December 11, 2020, divided by the number of students enrolled multiplied by 500.

^†^ GDPH and Georgia Department of Education contacted all public Georgia K–5 superintendents (1,321 schools) and private school leaders (140 schools); 169 schools with available case data completed the survey (response rate 11.6%).

^§^ Number includes both students and staff members with a case of COVID-19 during the study period.

^¶^ All RR estimates except for county COVID-19 incidence were adjusted for county-level 7-day case incidence per 100,000 population on December 1, 2020. RRs that exclude 1 are statistically significant.

** Per 100,000 population. County incidence was calculated as the 7-day cumulative sum of COVID-19 cases reported to GDPH on December 1, 2020, divided by the county population multiplied by 100,000. Population estimates for 2019 were provided by the Annual Estimates of the Resident Population for Counties in Georgia from April 1, 2010, to July 1, 2019.

^††^ Based on the 2013 National Center for Health Statistics classification. Metro counties include large metro (county population ≥1,000,000), medium metro (250,000–999,999), and small metro (<250,000); nonmetro counties include: micropolitan (10,000–49,999) and noncore (nonmetropolitan counties that did not qualify as micropolitan).

^§§^ For schools that are 100% in-person, students attend in-person for the full school week; for hybrid models, a combination of in-person and remote learning occurs on an alternating schedule.

^¶¶^ Two schools had discordant mask requirements for teachers and other staff members (i.e., one school required mask use among teachers, but not other staff members, and one school required mask use among other staff members, but not teachers). These were excluded from the calculation of the RR for mask requirements for teachers and staff members. All other schools either required masks for both teachers and staff members or allowed for optional mask use among both groups.

*** Includes schools that reported “No” to improving ventilation and six schools that reported decreasing room occupancy as the only ventilation improvement.

^†††^ Small groups of students who stay together throughout the day during in-person learning.

Prevention strategies implemented at participating schools included requiring masks for teachers and staff members (65.1%) or students (51.5%), flexible medical leave for teachers (81.7%), improved ventilation (51.5%), spacing all desks ≥6 ft apart (18.9%), and using barriers on all desks (22.5%). Schools reported a median of 9.0 (IQR = 8.0–9.0) locations with handwashing stations (Table 1).

During the 26 days from November 16 through December 11, 2020, participating schools reported a median of two COVID-19 cases (range = 0–15); COVID-19 incidence for all schools combined was 3.08 cases among students and staff members per 500 enrolled students. Community incidence in counties with participating schools during the same period was 1,055 per 100,000 persons of all ages, or approximately 5.28 per 500 population.^††††^ Mask requirements for teachers and staff members (RR = 0.63) and improved ventilation (RR = 0.61) were associated with lower incidence (Table 1). Among 123 schools that reported on ventilation improvements, dilution methods (opening doors, opening windows, or using fans) alone (RR = 0.65), or in combination with filtration (installation of HEPA filters) with or without purification (installation of UVGI) (RR = 0.52) were associated with lower COVID-19 incidence (Table 2).

**TABLE 2:** COVID-19 incidence* and rate ratios in 123 elementary schools,**^†^** by type of ventilation improvement as a COVID-19 prevention strategy — Georgia, November 16–December 11, 2020

Ventilation improvement	No. (%) of schools	No. of enrolled students	No. of cases^§^	Cases per 500 students enrolled (95% CI)	RR^¶^ (95% CI)
**Total**	**123 (100)**	**66,499**	**417**	**3.13 (2.84–3.44)**	**—**
None**	37 (30.1)	21,844	183	4.19 (3.63–4.84)	Ref
Dilution only^††^	39 (31.7)	21,562	127	2.94 (2.48–3.50)	0.65 (0.43–0.98)
Filtration ± purification only^§§^	16 (13.0)	9,133	45	2.46 (1.84–3.29)	0.69 (0.40–1.21)
Dilution and filtration ± purification^¶¶^	31 (25.2)	13,960	62	2.22 (1.73–2.84)	0.52 (0.32–0.83)

**Abbreviations:** CI = confidence interval; GDPH = Georgia Department of Public Health; HEPA = high-efficiency particulate absorbing; RR = rate ratio; UVGI = ultraviolet germicidal irradiation; ± = with or without.

* Case incidence in schools was calculated as the sum of cases reported to GDPH during November 16–December 11, 2020, divided by the number of students enrolled multiplied by 500.

^†^ Excludes schools from the original 169 that reported “Don’t know” to improving ventilation (n = 45) and one school that reported only using an air purification strategy.

^§^ Number includes both students and staff members with a case of COVID-19 during the study period.

^¶^ Adjusted for county-level 7-day case incidence per 100,000 population on December 1, 2020.

** Includes schools that reported “No” to improving ventilation and six schools that reported decreasing room occupancy as the only ventilation improvement.

^††^ Opening doors, opening windows, or using fans.

^§§^ Using HEPA filters with or without using UVGI and not opening doors, opening windows, or using fans.

^¶¶^ Opening doors, opening windows, or using fans, and using HEPA filters with or without using UVGI.

## Discussion

During November 16–December 11, 2020, many K–5 schools in Georgia had resumed in-person instruction,^§§§§^ necessitating implementation of strategies to prevent SARS-CoV-2 transmission within schools, including mask use and improved ventilation. This study found that before the availability of COVID-19 vaccines, the incidence of COVID-19 was 37% lower in schools that required mask use among teachers and staff members and was 39% lower in schools that reported implementing one or more strategies to improve classroom ventilation. Preventing transmission of SARS-CoV-2 in schools should be multifaceted (*2*). Mask requirements for teachers and staff members and improved ventilation are important strategies that elementary schools could implement as part of a multicomponent approach to provide safer, in-person learning environments until vaccines are available for children aged <12 years. 

CDC recommends implementing multiple prevention strategies (*2*) (e.g., physical distancing, masking, improved ventilation, and contact tracing) that have been associated with lower SARS-CoV-2 transmission in kindergarten through grade 12 settings (*3*–*5*). Since the completion of this study, COVID-19 vaccines have become widely available, and CDC recommends vaccination for teachers, staff members, and students aged ≥12 years (*2*). Until vaccines are available for children aged <12 years, universal and correct mask use is a critical prevention strategy CDC recommends that schools prioritize regardless of vaccination status for in-person learning (*2*). In the current study, the lower incidence in schools requiring mask use among teachers and staff members is consistent with research on mask effectiveness (*6*), and investigations that have identified school staff members as important contributors to school-based SARS-CoV-2 transmission (*7*). The 21% lower incidence in schools that required mask use among students was not statistically significant compared with schools where mask use was optional. This finding might be attributed to higher effectiveness of masks among adults, who are at higher risk for SARS-CoV-2 infection but might also result from differences in mask-wearing behavior among students in schools with optional requirements. Mask use requirements were limited in this sample; 65.1% of schools required teacher and staff member mask use and approximately one half (51.5%) required student mask use. Because universal and correct use of masks can reduce SARS-CoV-2 transmission (*6*) and is a relatively low-cost and easily implemented strategy, findings in this report suggest universal and correct mask use is an important COVID-19 prevention strategy in schools as part of a multicomponent approach.

In schools that improved ventilation through dilution methods alone, COVID-19 incidence was 35% lower, whereas in schools that combined dilution methods with filtration, incidence was 48% lower. Ventilation can be improved in simple, cost-effective ways by keeping doors and windows open and using fans to increase air flow from open windows (*8*). In rooms that are difficult to ventilate or have an increased likelihood of being occupied by persons with COVID-19 (e.g., nurse’s office), installation of HEPA filters or UVGI should be considered (*8**,**9*). However, only approximately one half (51.5%, 87 of 169) of school representatives reported being sure that ventilation was improved in school classrooms, and 18.0% (31 of 169) reported that their school implemented dilution methods in combination with filtration. These findings suggest that there are opportunities for many schools to reduce SARS-CoV-2 transmission through improved ventilation. Schools in lower-resourced communities might face barriers to installation of air filtration and purification devices; however, improvements can be made through dilution methods alone. CDC recommends improving ventilation through dilution, filtration, and purification methods, consistent with the school’s safety protocols (*8*).

The findings in this report are subject to at least four limitations. First, many COVID-19 cases were self-reported by staff members and parents or guardians, and prevention strategies reported by administrators or nurses might not reflect day-to-day activities or represent all school classrooms, and did not include an assessment of compliance (e.g., mask use). Second, the study had limited power to detect lower incidence for potentially effective, but less frequently implemented strategies, such as air filtration and purification systems; only 16 schools reported implementing this ventilation improvement. Third, the response rate was low (11.6%), and some participating schools had missing information about ventilation improvements. However, incidence per 500 students was similar between participating (3.08 cases) and nonparticipating (2.90 cases) schools, suggesting any systematic bias might be low. Finally, the data from this cross-sectional study cannot be used to infer causal relationships.

This study highlighted the importance of masking and ventilation for preventing SARS-CoV-2 transmission in elementary schools and revealed important opportunities for increasing their use among schools. A multicomponent approach to school COVID-19 prevention efforts is recommended (*2*), and requirements for universal and correct mask use among teachers and staff members and improved ventilation are two important strategies that could reduce SARS-CoV-2 transmission as schools continue, or return to, in-person learning.

**Summary**

**What is already known about this topic?**

Kindergarten through grade 5 schools educate and address the students’ physical, social, and emotional needs. Preventing SARS-CoV-2 transmission in schools is imperative for safe in-person learning.

**What is added by this report?**

COVID-19 incidence was 37% lower in schools that required teachers and staff members to use masks and 39% lower in schools that improved ventilation. Ventilation strategies associated with lower school incidence included dilution methods alone (35% lower incidence) or in combination with filtration methods (48% lower incidence).

**What are the implications for public health practice?**

Mask requirements for teachers and staff members and improved ventilation are important strategies in addition to vaccination of teachers and staff members that elementary schools could implement as part of a multicomponent approach to provide safer, in-person learning environments.

## Footnotes

* Ventilation strategies include dilution methods (opening doors, opening windows, and using fans to improve circulation from open windows); filtration methods (installation of high-efficiency particulate absorbing [HEPA] filters); and purification methods (installation of ultraviolet germicidal irradiation [UVGI] units, installed in upper room areas and shielded from persons or installed in the heating, ventilation, and air conditioning [HVAC] system). https://www.cdc.gov/coronavirus/2019-ncov/community/schools-childcare/ventilation.html

†
https://www.cdc.gov/coronavirus/2019-ncov/community/schools-childcare/operation-strategy.html

§ This denominator closely represents the size of elementary schools included in this study (median = 532).

¶ COVID-19 cases among staff members and students are defined as laboratory-confirmed reverse transcription–polymerase chain reaction or rapid antigen positive test results self-reported to the school by staff members and parents or guardians of students or by local public health officials. Schools report aggregate counts of cases among students and staff members weekly to GDPH and are required to report even if they have no cases.

** Public school; public charter, magnet, or alternative school; private, parochial, or independent school.

†† Based on the 2013 National Center for Health Statistics classification. Metro counties include large metro (county population ≥1,000,000), medium metro (250,000–999,999), and small metro (<250,000); nonmetro counties include micropolitan (10,000–49,999) and noncore (nonmetropolitan counties that did not qualify as micropolitan).

§§ For schools that are 100% in-person, students attend in-person for the full school week; for hybrid models, a combination of in-person and remote learning occurs on an alternating schedule.

¶¶ White, African American or Black, Hispanic, Asian, American Indian or Alaska Native, Other Pacific Islander, and Multiracial.

*** Schools reported “Yes” or “No” to the question, “Are steps being taken to improve air quality and increase the ventilation in the school?” Schools that responded “Yes” were asked to select from the following options: opening doors, opening windows, using fans to increase effectiveness of open windows, installation of HEPA filtration systems in high-risk areas, or installation of UVGI in high-risk areas. Multiple choices were allowed.

††† County incidence was calculated as the 7-day cumulative sum of COVID-19 cases reported to GDPH on December 1, 2020, divided by the county population multiplied by 100,000. Population estimates for 2019 were provided by the Annual Estimates of the Resident Population for Counties in Georgia from April 1, 2010, to July 1, 2019. Data were obtained from the U.S. Census Bureau on October 1, 2020.

§§§ 45 C.F.R. part 46, 21 C.F.R. part 56; 42 U.S.C. Sect. 241(d); 5 U.S.C. Sect. 552a; 44 U.S.C. Sect. 3501 et seq.

¶¶¶ “Available case data” refers to the weekly aggregate COVID-19 case reports provided by schools to GDPH. Not all schools that completed the survey reported during the study period. Sixty-one schools that completed the survey but did not provide GDPH any weekly COVID-19 reports during the study period were excluded.

**** Median proportions of American Indian or Alaska Native and Native Hawaiian or Other Pacific Islander were <1%. Each school reported the proportion of students who identified within the different racial and ethnic groups. The cumulative proportions could not exceed 100%.

†††† Community incidence was calculated for the survey period November 16–December 11, 2020, to allow comparison to school-level incidence during the same period. County-level incidence used for RR estimation and the figure are 7-day cumulative cases per 100,000 population as reported on December 1, 2020.

§§§§ Based on data reported to GDPH as part of COVID-19 surveillance in schools.
